# A quantitative analysis of the reduction in oxygen levels required to induce up-regulation of vascular endothelial growth factor (VEGF) mRNA in cervical cancer cell lines

**DOI:** 10.1038/sj.bjc.6690555

**Published:** 1999-07

**Authors:** J A Chiarotto, R P Hill

**Affiliations:** Experimental Therapeutics Division, Research Department, Ontario Cancer Institute/Princess Margaret Hospital, and Department of Medical Biophysics, University of Toronto, 610 University Avenue, Toronto, Ontario, Canada M5G 2M9

**Keywords:** VEGF, hypoxia, oxygen concentration, cervical cancer, gene up-regulation

## Abstract

The presence of hypoxia (low oxygen concentrations) in solid tumours correlates with poor prognosis, increased metastasis, and resistance to radiotherapy and some forms of chemotherapy. Malignant cells produce an angiogenesis factor, vascular endothelial growth factor (VEGF), which may increase metastatic ability and is up-regulated in the presence of hypoxia. Clinical data for cancers of the cervix and head and neck relate oxygen levels in the tumour to treatment outcome. This suggests the possibility that the presence of VEGF mRNA might be used as a marker for relevant levels of hypoxia. Suspension cultures of three human cervical cancer cell lines, SiHa, ME-180 and HeLa, were used to investigate up-regulation of VEGF mRNA levels following exposure to precisely defined oxygen concentrations for 2 or 4 h. An oxygen sensor was used to confirm the actual levels of dissolved oxygen present. The oxygen concentrations which caused half-maximal upregulation (the *K*_m_ value) of VEGF mRNA level in the three cell lines were similar except for one instance (*K*_m_ at 4 h: SiHa 27.0 ± 5.7 μM, ME-180 16.8 ± 3.3 μM, HeLa 13.0 ± 1.8 μM, SiHa and HeLa *P* = 0.01). The *K*_m_ values for the HeLa cell line as measured at 2 h (24.9 ± 0.8 μM) and 4 h (13.0 ± 1.8 μM) were significantly different (*P* < 0.0001). VEGF mRNA half-lives measured in air were consistent with values in the literature (SiHa 59.8 ± 5.8 min, ME-180 44.4 ± 7.2 min, HeLa 44.5 ± 6.3 min). Differences in oxygen consumption at low oxygen concentrations were noted between the different cell lines. Stirring in suspension culture was found to induce VEGF mRNA in SiHa cells. The presence of VEGF mRNA may be a marker for radiobiologic hypoxia. © 1999 Cancer Research Campaign


*British Journal of Cancer* (1999) **80**(10), 1518–1524
© 1999 Cancer Research Campaign
Article no. bjoc.1999.0555

### A quantitative analysis of the reduction in oxygen levels required to induce up-regulation of vascular endothelial growth factor (VEGF) mRNA in cervical cancer cell lines

**JA Chiarotto and RP Hill**

Experimental Therapeutics Division, Research Department, Ontario Cancer Institute/Princess Margaret Hospital, and Department of Medical Biophysics, University of Toronto, 610 University Avenue, Toronto, Ontario, Canada M5G 2M9

**Summary** The presence of hypoxia (low oxygen concentrations) in solid tumours correlates with poor prognosis, increased metastasis, and resistance to radiotherapy and some forms of chemotherapy. Malignant cells produce an angiogenesis factor, vascular endothelial growth factor (VEGF), which may increase metastatic ability and is up-regulated in the presence of hypoxia. Clinical data for cancers of the cervix and head and neck relate oxygen levels in the tumour to treatment outcome. This suggests the possibility that the presence of VEGF mRNA might be used as a marker for relevant levels of hypoxia. Suspension cultures of three human cervical cancer cell lines, SiHa, ME-180 and HeLa, were used to investigate up-regulation of VEGF mRNA levels following exposure to precisely defined oxygen concentrations for 2 or 4 h. An oxygen sensor was used to confirm the actual levels of dissolved oxygen present. The oxygen concentrations which caused half-maximal upregulation (the $K_m$ value) of VEGF mRNA level in the three cell lines were similar except for one instance ($K_m$ at 4 h: SiHa 27.0 ± 5.7 μM, ME-180 16.8 ± 3.3 μM, HeLa 13.0 ± 1.8 μM, SiHa and HeLa $P = 0.01$). The $K_m$ values for the HeLa cell line as measured at 2 h (24.9 ± 0.8 μM) and 4 h (13.0 ± 1.8 μM) were significantly different ($P < 0.0001$). VEGF mRNA half-lives measured in air were consistent with values in the literature (SiHa 59.8 ± 5.8 min, ME-180 44.4 ± 7.2 min, HeLa 44.5 ± 6.3 min). Differences in oxygen consumption at low oxygen concentrations were noted between the different cell lines. Stirring in suspension culture was found to induce VEGF mRNA in SiHa cells. The presence of VEGF mRNA may be a marker for radiobiologic hypoxia.

**Keywords:** VEGF; hypoxia; oxygen concentration; cervical cancer; gene up-regulation

Regions of hypoxia, or low oxygen tension, are known to exist within tumours (Raleigh et al, 1996; Brown and Giaccia, 1998; Dewhirst, 1998). Since radiotherapy and some forms of chemotherapy are less effective at killing cancer cells in hypoxic environments, much effort has been directed toward identifying tumours containing such regions. Measurement of oxygen tension by needle electrodes in lymph node metastasis of cancer of the head and neck found that radiation was less effective at inducing regression when the lymph nodes were hypoxic (Gatenby et al, 1988) and such measurements have been reported to identify patients with poor locoregional tumour control (Nordsmark et al, 1996). Similar work in advanced cancer of the uterine cervix (Hockel et al, 1993, 1996; Fyles et al, 1998; Hockel and Vaupel, 1998) showed that increased levels of hypoxia in the primary tumour mass correlated with poorer treatment outcome. These results suggested that hypoxia in cervical cancers correlated with a greater likelihood of both local failure and nodal metastasis. Metastases were also found to be more frequent in patients with the most hypoxic soft tissue sarcoma of the extremities (Brizel et al, 1996). Furthermore, exposure of cancer cells to hypoxia (Young et al, 1988; Jang and Hill, 1997) and hypoxia-induced increases in vascular endothelial growth factor (VEGF) secretion have been associated with an increased metastatic ability (Danielsen and Rofstad, 1998).

VEGF is the most selective vascular endothelial cell mitogen known (Dvorak et al, 1995). In cancer cells, the four VEGF isoforms which have been most frequently described contain 121, 165, 189 and 206 amino acids (Tischer et al, 1991). In some studies, increased intra-tumoural VEGF mRNA and protein has been associated with poor prognosis (Berger et al, 1995; Toi et al, 1995), increased metastasis (Brown et al, 1995; Takahashi et al, 1995), and increased microvessel density (Guidi et al, 1995; Toi et al, 1995; Fontini et al, 1997). Antibodies directed towards VEGF can inhibit angiogenesis and the proliferation of cancer cells in vivo (Kim et al, 1993; Kondo et al, 1993). Cancer cells constitutively produce VEGF (Dvorak et al, 1995) and can up-regulate its expression under hypoxic stress (Shweiki et al, 1992) via the transcription factor hypoxia-inducible factor 1 (Forsythe et al, 1996) and by stabilization of the mRNA (Levy et al, 1996, 1998).

The qualitative relationship between oxygen level and VEGF up-regulation has been examined in a variety of different tumour systems but has not been quantitatively documented in most of the studies performed (Shweiki et al, 1992; Minchenko et al, 1994; Leith and Michelson, 1995; Mukhapadhyay et al, 1995). The purpose of the present work was to examine the relationship between VEGF mRNA level and oxygen concentration in detail and to determine the extent of its variation between different tumour cells of similar histopathological type. Cell lines derived from human cancer of the uterine cervix were chosen for the study based on the abundance of clinical data relating tumour

*Received 21 September 1998*
*Revised 28 January 1999*
*Accepted 2 February 1999*

*Correspondence to:* RP Hill oxygenation as measured by the Eppendorf $pO_2$ Histograph to treatment outcome and the possibility that VEGF mRNA might be usable as a marker for relevant levels of hypoxia in such tumours. VEGF mRNA rather than protein was chosen for study because it is localized to the cell which is under hypoxic stress and is thus able to localize this environment.

### MATERIALS AND METHODS

### Cells

Cell lines used in the experiments were SiHa, ME-180, and HeLa which are derived from human cancer of the uterine cervix. The SiHa and ME-180 cells were obtained from ATCC (American Type Culture Collection, Manassas, VA, USA), while the HeLa cells were obtained from the laboratory of Dr Michael Rauth at OCI/PMH where they had been grown for many years. These cells were grown in plastic tissue culture flasks (Gibco BRL, Burlington, ON, Canada) in α-minimal essential medium (α-MEM; Gibco BRL, Burlington, ON, Canada) plus 10% fetal bovine serum (FBS; Wisent, Quebec, Canada) plus antibiotics for SiHa and HeLa and in McCoy's 5A medium plus 10% FBS plus antibiotics for ME-180. The cells were grown to about 70% confluence, then trypsinized, counted and a volume containing $3 \times 10^6$ cells was spun down at 130 ***g*** at 4°C. The supernatant was then poured off, leaving a cell pellet which was resuspended in the remaining medium, approximately 50 µl, and introduced into the vials within 10 min of centrifugation as described below.

### Oxygenation

The apparatus used for accurate control of the level of oxygen exposure of the cells has been fully described (Whillans and Rauth, 1980). Briefly, the apparatus consisted of a 37°C water bath into which a set of glass vials, each containing a small magnetic stir bar and 10 ml of medium, was placed. The medium was stirred at 200 rpm and humidified gas was flowed through an inlet in the stopper for 90 min to achieve an equilibrium between the oxygen ($O_2$) in the gas phase and the liquid medium. The small volume of medium containing the cells was introduced into each of the vials (final concentration: $3 \times 10^5$ cells $ml^{-1}$) by sterile Pasteur pipet after the 90-min gassing period and the gassing and stirring was continued. At defined time intervals later, a vial was removed from the 37°C water bath and placed on ice prior to total RNA extraction. Gases containing $O_2$ concentrations of 21% (air), 6.25%, 4.85%, 3.46%, 2.11%, 1.57%, 1.00%, or 0% (< 10 ppm) each with 5% carbon dioxide ($CO_2$) and balance $N_2$ were used. The composition of the gases was analysed to be within 2% or better of the value given by the supplier (Praxair, Toronto, ON, Canada) and was confirmed in our laboratory.

Each cell line was checked twice for the occurrence of cellular aggregation after 4 h of stirring. No cellular aggregates were seen. The effect of the stirring on the cells was checked after 4 h of stirring in both $N_2$ and air environments by plating the cells in tissue culture dishes for colony formation. Plating efficiencies ranged from 50 to 80% of that found for cells which were not stirred (data not shown). The pH of the stirred cell suspension was checked for each cell line and was found to be unchanged at about 7.5 throughout the length of a 4-h gassing period.

### Oxygen measurements

Since the cells can be expected to consume some of the oxygen and hence influence the level of oxygen to which they are exposed, a Clark-type polarographic electrode (Marshall et al, 1986) was used to measure $O_2$ concentration in the cell-containing medium. A glass vial and stopper, into which an extra hole was cut to accommodate the oxygen sensor, was prepared as above. The sensor was first calibrated for $O_2$ concentration using the above series of gases. A total of $3 \times 10^6$ cells was introduced into stirred medium, as described above, and the resulting $O_2$ concentration was measured once a stable signal indicating equilibration had been achieved, a process requiring approximately 5 min. Results obtained for the different mixtures are shown in Table 1.

### RNAase protection assay

After exposure to gassing, the cells were spun into a pellet at 200 ***g*** at 4°C for 5 min, resuspended in phosphate-buffered saline (PBS), and repelleted as before. Total RNA was then extracted with Trizol (Gibco BRL, Burlington, ON, Canada) using the manufacturer's protocol and stored at –70°C until analysis. An RNAase protection assay was used to quantitate VEGF and 36B4 mRNA levels (Zinn et al, 1983). The 36B4 mRNA codes for an acidic ribosomal

**Table 1** Oxygen concentration in µM measured in medium containing $3 \times 10^5$ cells $ml^{-1}$ of one of the cell lines compared to the oxygen tension in the overlying gas in mmHg and to the expected oxygen concentration in the medium in the absence of cells in µM

| Ambient oxygen tension (mmHg) [% $O_2$ in gassing mixture] | Expected oxygen concentration (µM) | SiHa cells ± s.e.m. (µM) | ME-180 cells ± s.e.m. (µM) | HeLa cells ± s.e.m. (µM) |
|---|---|---|---|---|
| 7.5 [1.00] | 10.6 | 2.1 ± 0.7[a] | 5.5 ± 1.3[a] | 6.4 ± 1.0[a] |
| 11.2 [1.57] | 15.9 | 8.9 ± 1.1 | 10.2 ± 1.4 | 10.8 ± 0.6 |
| 15.1 [2.11] | 21.4 | 16.5 ± 0.8 | 14.9 ± 1.6 | 16.8 ± 0.4 |
| 24.8 [3.46] | 35.2 | 30.7 ± 2.1 | 27.1 ± 0.8 | 28.5 ± 2.4 |
| 34.7 [4.85] | 49.3 | 46.1 ± 5.1 | – | 40.3 ± 1.8 |
| 44.8 [6.25] | 63.6 | 59.5 ± 1.4 | 53.7 ± 2.0 | 52.0 ± 1.4 |
| 141.7 [air] | 200.3 | 194.3 ± 3.8 | 196.0 ± 5.4 | 192.6 ± 4.3 |

[a]Oxygen concentration in medium containing SiHa cells significantly different from medium containing ME-180 cells ($P = 0.01$) or HeLa cells ($P < 0.001$).

© 1999 Cancer Research Campaign 

protein (Laborda, 1991) and served as a loading control. Briefly, riboprobes were purified on a 6% polyacrylamide/urea gel then eluted overnight at 37°C in elution buffer (0.5 M EDTA, 0.1% sodium dodecyl sulphate (SDS), 0.1 M EDTA), precipitated and their radioactivity quantified. The probes were hybridized in excess to 10 μg of total RNA overnight at 52°C. Samples were digested with 40 μg ml$^{-1}$ of RNAase and 2 μg ml$^{-1}$ of RNAase T1 for 30 min at 30°C. Ten microlitres of 20% SDS and 50 μg of proteinase K were then added, followed by incubation for 15 min at 37°C, phenol–chloroform extraction, and ethanol precipitation with glycogen. The protected probes were then resolved on a 6% polyacrylamide/urea gel and quantitated on a PhosphorImager (Molecular Dynamics, Sunnyvale, CA, USA).

### Probes

A probe for detecting VEGF mRNA was generated using the *Sty*I fragment (nucleotides 99–352) (Tischer et al, 1991) obtained from the cDNA for VEGF165 (kind gift of Dr Keith Laderoute, Stanford Research Institute). The fragment was blunted and subcloned into the *Sma*I site of the pBluescript II KS(−) cloning vector, which was then linearized with *Xba*I. Correct orientation of the insert was confirmed with the production of a 121-bp *Dra*II fragment. The $^{32}$P-radiolabelled antisense riboprobe, containing 253 nucleotides was capable of recognizing all four cancer-related isoforms. It was transcribed using a T7 RNA polymerase.

A cloning vector containing the cDNA for 36B4 (Laborda, 1991) (kind gift of Dr Linda Penn, Ontario Cancer Institute) was linearized with *Eco*RV. The $^{32}$P-radiolabelled antisense riboprobe containing 63 nucleotides, was transcribed using an SP6 RNA polymerase. For all the results presented, the amount of VEGF mRNA was normalized to the amount of 36B4 mRNA detected in the same lane on the polyacrylamide gel. A comparison of the 36B4 levels in cells gassed with 95% $N_2$–5% $CO_2$ for 0 h or 4 h, collected over 48 experiments for all three cell lines, showed no evidence of an effect of hypoxic exposure on the expression level (paired *t*-test, $P = 0.69$).

### Statistical analysis

The curves for VEGF mRNA upregulation as a function of oxygen concentration were fitted to a logistic function using the Levenburg–Marquardt algorithm in the Origin 5.1 software package (Microcal, Northampton, MA, USA) as described in the text. The $K_m$ values for VEGF mRNA up-regulation and the VEGF mRNA half-lives were compared in a two-tailed test using the Z statistic. Comparisons of oxygen concentrations in cell-containing medium were made using a two-tailed Student's *t*-test. In both cases significance was determined by a *P*-value less than 0.05.

### RESULTS

### Net increase in VEGF mRNA levels under long-term anoxia

Initially we examined the time course of the increase in VEGF mRNA levels during exposure to anoxia (< 10 ppm $O_2$). Cells were placed in a vial containing medium which had been equilibrated either with $N_2$–5% $CO_2$ (anoxic) or air–5% $CO_2$. The cells were then exposed to this environment for up to 12 h with samples

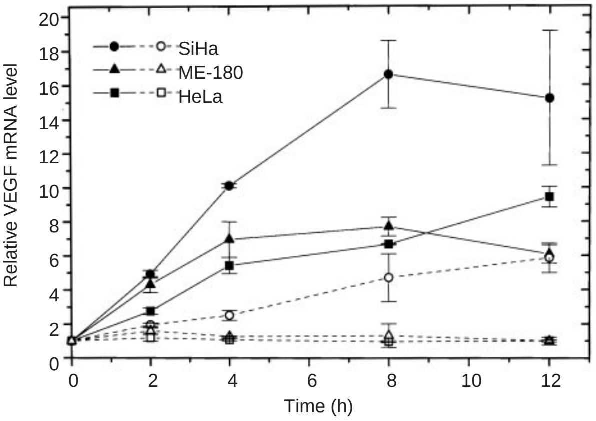

**Figure 1** Relative VEGF mRNA level in cells exposed to anoxia or air in stirred cell suspension as a function of time. Solid symbols with solid lines indicate exposure to anoxia. Open symbols with dashed lines indicate exposure to air. The absolute ratio of VEGF mRNA to 36B4 mRNA at time 0 h (mean ± s.e.m.) is 3.3 ± 0.3 for SiHa cells, 3.0 ± 0.3 for ME-180 cells, and 1.4 ± 0.1 for HeLa cells taken at 0, 2, 4, 8 and 12 h for analysis of mRNA levels. Figure 1 shows the results for each cell line under anoxic and air conditions. The degree of VEGF up-regulation was normalized to time 0 h for each cell line. The points represent the mean (± s.e.m.) of at least three independent experiments. By 4 h, VEGF mRNA in ME-180 cells had reached its maximum level. The HeLa line showed a gradual up-regulation of VEGF mRNA over the 12 h; however, the degree of up-regulation is similar to that in ME-180 cells. In both these cell lines only slight changes in VEGF mRNA levels occurred over 12 h under air conditions. In the SiHa cells the VEGF mRNA level reached a plateau after 8 h of anoxia. However, there was also an increase in the control air conditions particularly after 2 h, presumably as a result of the stress of being in the stirred suspension. Thus, a plateau for SiHa cells likely occurs earlier than 8 h.

### Analysis of VEGF mRNA levels in cells exposed to different oxygen concentrations

This set of experiments was performed to determine in detail the range of oxygen concentration over which VEGF mRNA is up-regulated. Cells were gassed with various oxygen concentrations using groups of three vials, that included a vial which contained cells exposed to the oxygen concentration in question, a vial containing cells which were gassed with 95% $N_2$–5% $CO_2$ (the positive control), and a vial containing cells gassed with 95% air–5% $CO_2$ (the negative control). Since the results in Figure 1 suggest that much of the effect of the hypoxic exposure occurred in the first 4 h of gassing, the cells were sampled at time 0, 2 and 4 h after the start of the gassing and total RNA extracted. The ratio of VEGF mRNA to 36B4 mRNA at 2 and 4 h was normalized to the ratio at 0 h. For each of the three cell lines, measurements at each of the above oxygen concentrations were performed at least in triplicate.

Figure 2A shows the relative VEGF mRNA level as a function of oxygen concentration for ME-180 cells measured at 2 and 4 h. Figures 2B and 2C show the 2- and 4-h data for SiHa and HeLa cells respectively. The data were fitted to a logistic function and

 *© 1999 Cancer Research Campaign*

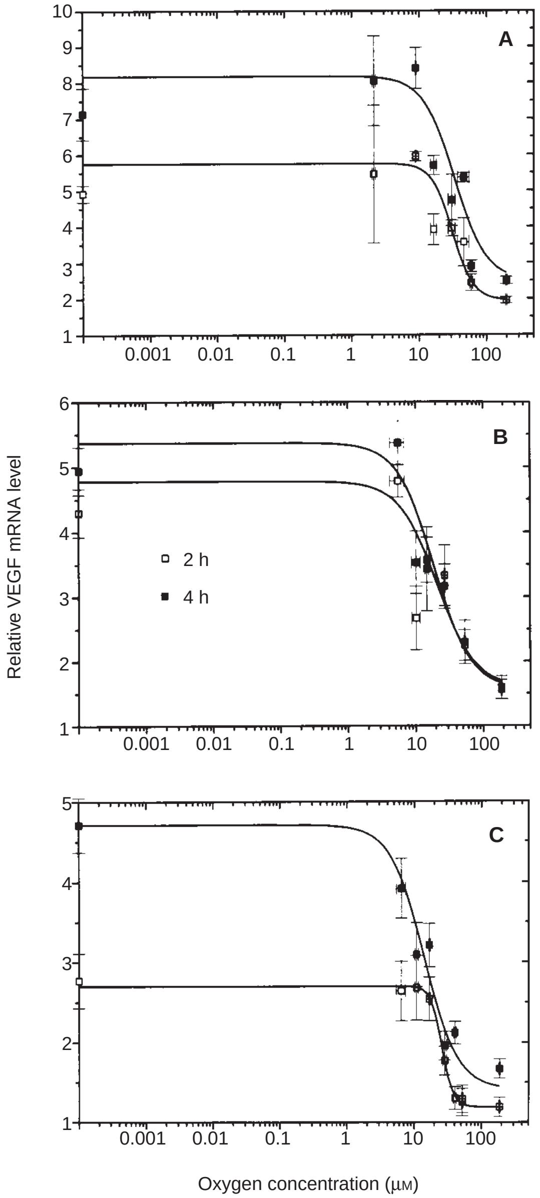

**Figure 2** Relative VEGF mRNA level as a function of oxygen concentration. Data for ME-180 cell line (**A**), SiHa cell line (**B**) and HeLa cell line (**C**) showing VEGF mRNA levels as measured at 2 h (□) and 4 h (■)

Table 2 Oxygen concentrations which cause half-maximal up-regulation of VEGF mRNA at 2 and 4 h and half-lives in presence of air

| Cell line | 2 h (µM ± 1 s.e.m.) | 4 h (µM ± 1 s.e.m.) | Half-life (min ± 1 s.e.m.) |
|---|---|---|---|
| SiHa | 29.1 ± 6.1 | 27.0 ± 5.7 | 59.8 ± 5.8 |
| ME-180 | 18.7 ± 6.9 | 16.8 ± 3.3 | 44.4 ± 7.2 |
| HeLa | 24.9 ± 0.7 | 13.0 ± 1.8 | 44.5 ± 6.3 |

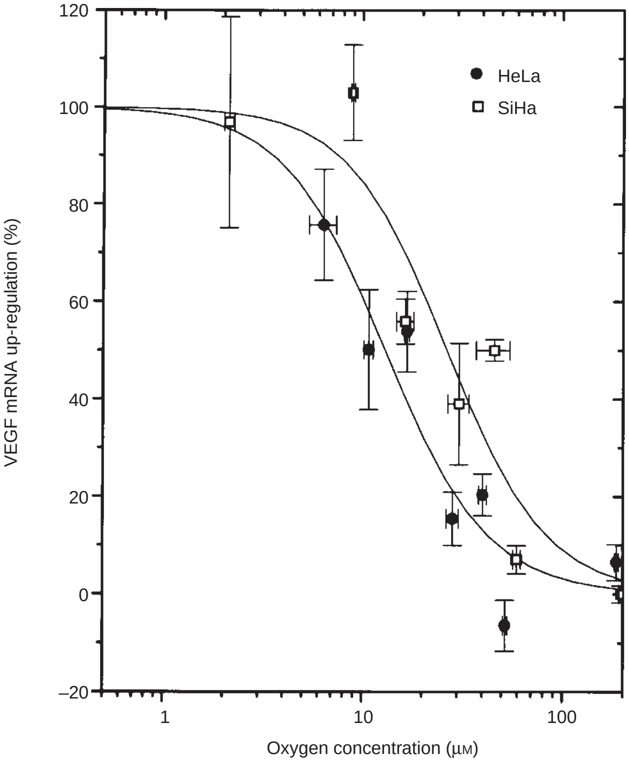

**Figure 3** Percentage change in VEGF mRNA level in the SiHa and HeLa cell lines as a function of oxygen concentration the oxygen concentration at which half-maximal up-regulation occurs was determined. During curve fitting, the maximum and minimum values were fixed based on observations of the data. The minimum values were the lowest points at the highest oxygen concentration with the single exception of 4-h data for the HeLa cells where an average of the values at the two highest oxygen concentrations was used. For the ME-180 cell line, the maximum level was chosen to be the highest single point (at 5.5 µM) in the 2-h and 4-h data set. In the case of the SiHa cells, the maximum value was an average of the two values (at 2.1 µM and 8.9 µM) which appeared to be on the upper plateau. For the SiHa and ME-180 cell lines, this calculation did not include the 95% $N_2$–5% $CO_2$ point because it was generally below the maximum value observed at intermediate oxygen levels and it is possible that a cell's ability to produce mRNA may be compromised at very low oxygen concentrations. This effect was not seen in the HeLa cell line, consequently for the HeLa cells, the maximum value was chosen to be the relative VEGF mRNA level at the anoxic point. Figure 2 indicates that the lines generated are generally a good fit to the data. Modifying these choices of maximum levels (or minimum levels for the HeLa 4-h data) modified slightly the calculated $K_m$ values but did not affect the conclusions drawn from the data.

The data shows that at high oxygen concentrations, i.e. above 60 µM, there is relatively little upregulation of VEGF mRNA. In the region between 10 µM and 50 µM, there is a dramatic increase in the amount of VEGF mRNA present in the cell. Below 10 µM, the up-regulation of VEGF mRNA appears to reach maximal levels. There may be a trend towards VEGF mRNA levels which are lower than maximal at very low oxygen levels, but this does not reach statistical significance.

Table 2 shows the oxygen concentrations which produce half-maximal upregulation at the 2- and 4-h time points. There were no statistical differences between the 2- and 4-h values for the SiHa and ME-180 cell lines. The difference between 2- and 4-h values for the HeLa cell line was significant ($P < 0.0001$) and remained

*© 1999 Cancer Research Campaign*

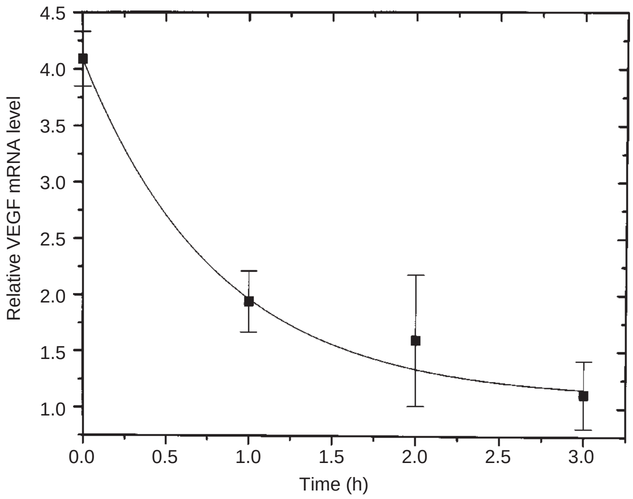

**Figure 4** Relative VEGF mRNA level in ME-180 cells exposed to anoxia for 3 h, then exposed to air as a function of time so with alternate choices for maximum and minimum. No statistical differences were seen among the 2-h values for the three cell lines. The 4-h value for the SiHa cell line and the HeLa cell line was statistically different ($P = 0.01$) and remained so with recalculation using alternate choices of maximum and minimum. Figure 3 illustrates this difference by showing the percentage change in VEGF mRNA levels in SiHa and HeLa cells at 4 h as a function of oxygen concentration. The data for ME180 cells at 4 h lies intermediate between these two curves and has been omitted for clarity.

### Determination of VEGF mRNA half-life in the presence of oxygen

In the final series of experiments we examined the stability of the VEGF mRNA when the cells were returned from anoxic exposure (< 10 ppm $O_2$) to exposure to air. The cells were placed in vials equilibrated with either 95% $N_2$–5% $CO_2$ or 95% air–5% $CO_2$, and stirred in their respective environments for 3 h, then one vial of each was sampled and the remaining 95% $N_2$–5% $CO_2$ vials were switched to gassing with 95% air–5% $CO_2$. The cell-containing medium required 4 min to reach an oxygen concentration of 100 μM at which point the effect of hypoxia on upregulation of VEGF mRNA was considered to be negligible. The vials were subsequently sampled at 1, 2 and 3 h. The level of VEGF mRNA in the $N_2$-gassed vials was divided by the values in the air controls and this ratio plotted against time. Figure 4 shows the results for ME-180 cells, fitted to an exponential decay curve. Similar results were obtained for SiHa and HeLa cells (data not shown). The half-life for reduction of the mRNA levels was determined for each cell line from the fitted exponential decay curve. The values obtained are shown in Table 2. These data suggest a similar decay time for all three cell lines. The calculated half-lives of the VEGF mRNA in ME-180 cells, HeLa cells and SiHa cells are consistent with values reported in the literature: approximately 40 min in rat glioblastoma (Stein et al, 1995) and 43 ± 6 min in rat pheochromocytoma cells (Levy et al, 1996).

### DISCUSSION

In the present study we examined the effect of different oxygen concentrations on the upregulation of VEGF mRNA in three cervical cancer cell lines. One aim of the experiments was to examine whether the presence of high levels of VEGF mRNA could serve as a surrogate marker for radiobiological hypoxia. A number of different cell lines were studied using the same techniques to determine if differences existed. VEGF mRNA was chosen for study over VEGF protein because the mRNA stays localized to the cell which is under hypoxic stress and is thus able to localize this environment.

Figure 1 shows that the rate of the hypoxia-induced increase in the relative level of VEGF mRNA differs amongst cell lines. To our knowledge, the increase in the relative level of VEGF mRNA associated with a stirred cell suspension, as was the case especially for the SiHa cell line, has not been described before. The physical stress of stirring, alteration of the cell shape while in suspension, or the loss of cell contact with its extracellular matrix may all contribute to this effect. A cancer cell may be exposed to similar stresses in the metastatic process. The increased vascular permeability caused by VEGF, partly due to the opening of endothelial intercellular junctions large enough to allow the passage of erythrocytes (Roberts and Palade, 1995), might allow a metastatic cell producing it to penetrate a microvascular wall more easily. The oxygen concentrations at which the VEGF mRNA is half-maximally up-regulated ($K_m$ value) appear to be cell line specific (Figure 2 and Table 2), indicating differences in the ability of cervical cancer cell lines to react to hypoxic stress. Differences in the sensitivity of the oxygen sensor within the cell, thought to be a haem protein (Bunn and Poyton, 1996), could explain these differences. One possible explanation could be the existence of intracellular oxygen gradients (Boag, 1970) which result in the oxygen concentration at the oxygen sensor being lower than that in the media surrounding the cells, thus triggering VEGF mRNA production at higher measured oxygen concentrations. At oxygen concentrations in the range of the $K_m$s for each of the cell lines the oxygen consumption appears to be equivalent (Table 1). However, at the lowest oxygen concentrations the SiHa cells appear to have a higher rate of oxygen consumption than HeLa and ME-180 cells (Table 1). Since the exact location of this sensor is not known (Bunn and Poyton, 1996), the significance of these differences cannot be assessed.

The observed shift in the $K_m$ value to a lower oxygen concentration at 4 h (vs 2 h) for the HeLa cell line may indicate a change in the cell line's ability to react to hypoxic stress with time.

The oxygen dependence of HIF-1 protein production and DNA-binding activity has been studied previously using the HeLa cell line (Jiang et al, 1996). The half-maximal value for these two activities, measured after 4 h of treatment, occurred between 1.5 and 2% oxygen (15–20 μM). This value is close to that observed for HeLa cells in the present study (13.0 ± 1.8 μM). In deriving their value, Jiang et al circumvented the problem of oxygen gradients created by cellular respiration by inhibiting oxidative phosphorylation with potassium cyanide (KCN). The presence of KCN altered the HIF-1 subunit levels, however the oxygen value associated with the half-maximal HIF-1 level in the presence or absence of KCN were about the same.

A number of other studies have shown up-regulation of VEGF mRNA with hypoxia, but in most cases have not examined its

© 1999 Cancer Research Campaign dependence on oxygen concentration quantitatively (Shweiki et al, 1992; Minchenko et al, 1994; Mukhapadhyay et al, 1995). To our knowledge only one study has examined the oxygen concentration dependence of VEGF production in cells (Leith and Michelson, 1995). In this study VEGF protein production was examined in two colon cancer cell lines exposed to a range of oxygen concentrations. Similar VEGF protein secretion rates were observed at oxygen concentrations in the gas phase below 0.3%. The apparatus for control of oxygen concentration consisted of cells in monolayer culture with an overlying 2.3-mm layer of medium over which flowed gas of accurately known oxygen concentration. With this depth of medium, a significant oxygen gradient due to cellular respiration would exist (Koch, 1984) and would result in uncertainty in the actual oxygen concentration to which the cells were exposed. In fact, the cell density was similar to that in the present study ($2.8 \times 10^5$ vs $3.0 \times 10^5$ cells $ml^{-1}$) where a decrease in oxygen concentration due to cellular respiration was clearly demonstrated (Table 1). The present study was conducted in a well-controlled environment with direct oxygen measurements and specifically measured the $K_m$ value for upregulation of the mRNA, not protein, and showed that differences in that value may exist in cells of the same cancer type.

Interestingly, the $K_m$ values that we have measured for VEGF up-regulation are similar to the oxygen concentrations which stimulate the ability of endothelial cells to form capillary networks (Helmlinger and Jain, 1998). They are also low enough that, if oxygen concentration were the only deteminant for VEGF production, most tumours should contain areas which are hypoxic enough (Vaupel and Hockel, 1998) to have large areas which stain for VEGF. In-situ hybridization which showed that VEGF mRNA was found primarily in cells adjacent to areas of necrosis (Shweiki et al, 1992) or tumour cell nests (Plate et al, 1994) did not attempt to quantitate the oxygen concentration within the tumour. To what extent the observed variation in VEGF staining for protein or mRNA in human tumours may reflect technical factors is not known (Senger et al, 1993; Guidi et al, 1995).

One possible use for a detailed understanding of the oxygen dependence of VEGF mRNA up-regulation would be as a marker for hypoxia, especially radiobiological hypoxia. Oxygen acts as a radiation sensitizer and the $K_m$ value for half maximum radiosensitization is usually regarded as being in the range of 2–5 mmHg (3–7 μM) (Chapman et al, 1974; Vaupel et al, 1989). However, recent studies in our laboratory with the SiHa and ME-180 cell lines used in this study suggest much higher values (Vukovic et al, 1998) similar to the $K_m$ values for VEGF mRNA upregulation reported here. Thus, such up-regulation may prove useful as a marker for radiobiologic hypoxia.

Physiologic conditions other than hypoxia are known to cause up-regulation of VEGF. Low glucose levels in the presence of oxygen (Shweiki et al, 1995) and low pH (Xie et al, 1998) can both increase VEGF levels in vitro. This complicates the interpretation of changes in the level of VEGF in vivo. The levels of hypoxia required for its upregulation in vivo need to be established. This might be done with the use of a marker for hypoxia such as the 2-nitroimidazole EF-5, which has a $K_m$ value for binding of about 1 mmHg (1.5 μM) (Koch et al, 1995). The amount of EF-5 bound in cells can be used to determine their oxygen level during exposure to EF-5 and seems to correlate with VEGF protein expression in spheroids (Waleh et al, 1995); however, it has been recently reported that pimonidazole binding does not correlate with VEGF protein expression in human squamous cell carcinomas (Raleigh et al, 1998).

Increased VEGF production by a cell may enhance its ability to form metastases. Evidence exists linking increased production of VEGF in rodent tumour cells (Jang and Hill, 1997), human melanoma cells (Claffey et al, 1996; Slaven et al, 1997; Danielsen and Rofstad, 1998), and human fibrosarcoma cells (Goldman et al, 1998) to increased ability to form metastases. Thus, tumours in which cells up-regulated VEGF at higher oxygen concentrations may be more likely to form metastases.

### ACKNOWLEDGEMENTS

We thank Dr. Keith Laderoute for his gift of the VEGF probe and Dr Linda Penn for her gift of the 36B4 probe and help with the RNAase protection assay. We thank Dr AM Rauth for his advice concerning the stirred cell apparatus, and both he and Dr A Fyles for their critical review of this manuscript. We thank Dr S Minkin for his assistance with statistical analysis and Anne Jang and Wilson Marhin for their help and technical expertise. This work was supported by a grant from the National Cancer Institute of Canada with funds raised by the Terry Fox Run.

### REFERENCES

Berger DP, Herbstritt L, Dengler WA, Marme D, Mertelsmann R and Fiebig HH (1995) Vascular endothelial growth factor (VEGF) mRNA expression in human tumor models of different histologies. *Ann Oncol* **6**: 817–825

Boag JW (1970) Cell respiration as a function of oxygen tension. *Int J Radiat Biol Relat Stud Phys Chem Med* **18**: 475–478

Brizel DM, Scully SP, Harrelson JM, Layfield LJ, Bean JM, Prosnitz LR and Dewhirst MW (1996) Tumor oxygenation predicts for the likelihood of distant metastases in human soft tissue sarcoma. *Cancer Res* **56**: 941–943

Brown JM and Giaccia AJ (1998) The unique physiology of solid tumors: opportunities (and problems) for cancer therapy. *Cancer Res* **58**: 1408–1416

Brown LF, Berse B, Jackman RW, Tognazzi K, Guidi AJ, Dvorak HF, Senger DR, Connolly JL and Schnitt SJ (1995) Expression of vascular permeability factor (vascular endothelial growth factor) and its receptors in breast cancer. *Hum Pathol* **26**: 86–91

Bunn HF and Poyton RO (1996) Oxygen sensing and molecular adaptation to hypoxia. *Physiol Rev* **76**: 839–885

Chapman JD, Dugle DL, Reuvers AP, Meeker BE and Borsa J (1974) Studies on the radiosensitizing effect of oxygen in Chinese hamster cells. *Int J Radiat Biol* **26**: 383–389

Claffey KP, Brown LF, del Aguila LF, Tognazzi K, Yeo K-T, Manseau EJ and Dvorak HF (1996) Expression of vascular permeability factor/vascular endothelial growth factor by melanoma cells increases tumor growth, angiogenesis, and experimental metastasis *Cancer Res* **56**: 172–181

Danielsen T and Rofstad EK (1998) Hypoxia-induced metastasis of human melanoma cells: involvement of vascular endothelial growth factor-mediated angiogenesis. *Forty-sixth Annual Meeting of the Radiation Research Society* 168

Dewhirst MW (1998) Concepts of oxygen transport at the microcirculatory level. *Semin Radiat Oncol* **8**: 143–150

Dvorak HF, Brown LF, Detmar M and Dvorak AM (1995) Vascular permeability factor/vascular endothelial growth factor, microvascular hyperpermeability, and angiogenesis. *Am J Pathol* **146**: 1029–1039

Fontini G, Vignati S, Lucchi M, Mussi A, Calcinai A, Boldrini L, Chine S, Silvestri V, Angeletti CA, Basolo F and Bevilacqua G (1997) Neoangiogenesis and p53 protein in lung cancer: their prognostic role and their relation with vascular endothelial growth factor (VEGF) expression. *Br J Cancer* **75**: 1295–1301

Forsythe JA, Jiang B-H, Iyer NV, Agani F, Leung SW, Koos RD and Semenza GL (1996) Activation of vascular endothelial growth factor gene transcription by hypoxia-inducible factor 1. *Mol Cell Biol* **16**: 4604–4613

Fyles AW, Milosevic M, Wong R, Kavanagh M-C, Pintilie M, Chapman W, Levin W, Manchul L, Keane TJ and Hill RP (1998) Oxygenation predicts radiation response and survival in patients with cervix cancer. *Radiother Oncol* **48**: 149–156

Gatenby RA, Kessler HB, Rosenbloom JS, Coia LR, Moldofsky PJ, Hartz WH and Broder GJ (1988) Oxygen distribution in squamous cell carcinoma metastases

© 1999 Cancer Research Campaign and its relationship to outcome of radiation therapy. *Int J Radiat Oncol Biol Phys* **14**: 831–838

Goldman CK, Kendall RL, Cabrera G, Soroceanu L, Heike Y, Gillespie GY, Siegal GP, Mao X, Bett AJ, Huckle WR, Thomas KA and Curiel DT (1998) Paracrine expression of a native soluble vascular endothelial growth factor receptor inhibits tumor growth, metastasis, and mortality rate. *Proc Natl Acad Sci USA* **95**: 8795–8800

Guidi AJ, Abu-Jawdeh G, Berse B, Jackman RW, Tognazzi K, Dvorak HF and Brown LF (1995) Vascular permeability factor (vascular endothelial growth factor) expression and angiogenesis in cervical neoplasia. *J Natl Cancer Inst* **87**: 1237–1244

Helmlinger G and Jain R (1998) Private communication

Hockel M, Knoop C, Schlenger K, Vorndran B, Baussman E, Mitze M, Knapstein PG and Vaupel P (1993) Intratumoral $pO_2$ predicts survival in advanced cancer of the uterine cervix. *Radiother Oncol* **26**: 45–50

Hockel M, Schlenger K, Aral B, Mitze M, Schaffer U and Vaupel P (1996) Association between tumor hypoxia and malignant progression in advanced cancer of the uterine cervix. *Cancer Res* **56**: 4509–4515

Hockel M and Vaupel P (1998) The prognostic significance of hypoxia in cervical cancer: a radiobiological or tumor biological phenomenon? In: *Blood Perfusion and Microenvironment of Human Tumors,* Molls M and Vaupel P (eds), pp. 73–79. Springer: New York

Jang A and Hill RP (1997) An examination of the effects of hypoxia, acidosis, and glucose starvation on the expression of metastasis-associated genes in murine tumor cells. *Clin Exp Metastas* **15**: 469–483

Jiang B-H, Semenza GL, Bauer C and Marti HH (1996) Hypoxia-inducible factor 1 levels vary exponentially over a physiologically relevant range of $O_2$ tension. *Am J Physiol* **271**: C1172–C1180

Kim KJ, Li B, Winer J, Armanini M, Gillett N, Phillips HS and Ferrara N (1993) Inhibition of vascular endothelial growth factor-induced angiogenesis suppresses tumour growth in vivo. *Nature* **362**: 841–844

Koch CJ (1984) A thin-film culturing technique allowing rapid gas-liquid equilibration (6 sec) with no toxicity to mammalian cells. *Radiat Res* **97**: 434–442

Koch CJ, Evans SM and Lord EM (1995) Oxygen dependence of cellular uptake of EF5 [2-(nitro-1H-imidazole-1-yl)-*N*-(2,2,3,3,3-pentafluoropropyl)acetamide]: analysis of drug adducts by fluorescent antibodies vs bound radioactivity. *Br J Cancer* **72**: 869–874

Kondo S, Asano M and Suzuki H (1993) Significance of vascular endothelial growth factor/vascular permeability factor for solid tumor growth, and its inhibition by the antibody. *Biochem Biophys Res Commun* **194**: 1234–1241

Laborda J (1991) 36B4 cDNA used as an estradiol-independent mRNA control is the cDNA for human acidic ribosomal phosphoprotein PO. *Nucleic Acids Res* **19**: 3998

Leith JT and Michelson S (1995) Secretion rates and levels of vascular endothelial growth factor in clones of HCT-8 human colon tumour cells as a function of oxygen concentration. *Cell Prolif* **28**: 415–430

Levy AP, Levy NS and Goldberg MA (1996) Post-transcriptional regulation of vascular endothelial growth factor by hypoxia. *J Biol Chem* **271**: 2746–2753

Levy NS, Chung S, Furneaux H and Levy AP (1998) Hypoxic stabilization of vascular endothelial growth factor mRNA by the RNA-binding protein HuR. *J Biol Chem* **273**: 6417–6423

Marshall RS, Koch CJ and Rauth AM (1986) Measurement of low levels of oxygen and their effect on respiration in cell suspensions maintained in an open system. *Radiat Res* **108**: 91–101

Minchenko A, Bauer T, Salceda S and Caro J (1994) Hypoxic stimulation of vascular endothelial growth factor expression in vitro and in vivo. *Lab Invest* **71**: 374–379

Mukhapadhyay D, Tsiokas L, Zhou X-MZ, Foster D, Brugge JS and Sukhatme VP (1995) Hypoxic induction of human vascular endothelial growth factor expression through c-Src activation. *Nature* **375**: 577–581

Nordsmark M, Overgaard M and Overgaard J (1996) Pretreatment oxygenation predicts radiation response in advance squamous cell carcinoma of the head and neck. *Radiother Oncol* **41**: 31–39

Plate KH, Brier G, Weich HA, Mennel HD and Risau W (1994) Vascular endothelial growth factor and glioma angiogenesis: coordinate induction of VEGF receptors, distribution of VEGF protein and possible in vivo regulatory mechanisms. *Int J Cancer* **59**: 520–529

Raleigh JA, Calkins-Adams DP, Rinker LH, Ballenger CA, Weissler MC, Fowler WC, Novotny DB, Weissler MC and Varia MA (1998) Hypoxia and vascular endothelial growth factor expression in human squamous cell carcinoma using pimonidazole as a hypoxia marker. *Cancer Res* **58**: 3765–3768

Raleigh JA, Dewhirst MW and Thrall DE (1996) Measuring tumor hypoxia. *Semin Radiat Oncol* **6**: 37–45

Roberts WG and Palade GE (1995) Increased microvascular permeability and endothelial fenestration induced by vascular endothelial growth factor. *J Cell Sci* **108**: 2369–2379

Senger DR, Van De Water L, Brown LF, Nagy JA, Yeo K-T, Yeo T-K, Berse B, Jackman RW, Dvorak AM and Dvorak HF (1993) Vascular permeability factor (VPF, VEGF) in tumor biology. *Cancer Metast Rev* **12**: 303–324

Shweiki D, Itin A, Soffer D and Keshet E (1992) Vascular endothelial growth factor induced by hypoxia may mediate hypoxia-initiated angiogenesis. *Nature* **359**: 843–848.

Shweiki D, Neeman M, Itin A and Keshet E (1995) Induction of vascular endothelial growth factor expression by hypoxia and by glucose deficiency in multicell spheroids: implications for tumor angiogeneis. *Proc Natl Acad Sci USA* **92**: 768–772

Slaven P, Heikkila P and Joensuu H (1997) Enhanced expression of vascular endothelial growth factor in metastatic melanoma. *Br J Cancer* **76**: 930–934

Stein I, Neeman M, Shweiki D, Itin A and Keshet E (1995) Stabilization of vascular endothelial growth factor mRNA by hypoxia and hypoglycemia and co-regulation with other ischemia-induced genes. *Mol Cell Biol* **15**: 5363–5368

Takahashi Y, Kitadai Y, Bucana CD, Cleary KR and Ellis LM (1995) Expression of vascular endothial growth factor and its receptor, KDR, correlates with vascularity, metastasis, and proliferation of human colon cancer. *Cancer Res* **55**: 3964–3968

Tischer E, Mitchell R, Hartman T, Silva M, Gospodarowicz D, Fiddes JC and Abraham JA (1991) The human gene for vascular endothelial growth factor. *J Biol Chem* **266**: 11947–11954

Toi M, Inada K, Suzuki H and Tominaga T (1995) Tumor angiogenesis in breast cancer: its importance as a prognostic indicator and association with vascular endothelial growth factor expression. *Breast Cancer Res Treat* **36**: 193–204

Vaupel P and Hockel M (1998) Oxygenation of human tumors In: *Blood Perfusion and Microenvironment of Human Tumors* Molls, M and Vaupel P (eds), pp. 63–72. Springer: New York

Vaupel P, Kallinowski F and Okunieff P (1989) Blood flow, oxygen and nutrient supply, and metabolic microenvironment of human tumors: a review. *Cancer Res* **49**: 6449–6465

Vukovic V, Hill RP, Rauth AM and Hedley DW (1998) Effects of BSO on NPSH profiles, DNA single strand breaks and cell survival in ME180 and SiHa cells irradiated at intermediate oxygen concentrations. *Radiat Res* (submitted)

Waleh NS, Brody MD, Knapp MA, Mendonca HL, Lord EM, Koch CJ, Laderoute KR and Sutherland RM (1995) Mapping of the vascular endothelial growth factor-producing hypoxic cell in multicellular tumor spheroids using a hypoxia-specific marker. *Cancer Res* **55**: 6222–6226

Whillans DW and Rauth AM (1980) An experimental and analytical study of oxygen depletion in stirred cell suspensions. *Radiat Res* **84**: 97–114.

Xie K, Haung S, Xu L and Fidler IJ (1998) Molecular mechanisms for the regulation of vascular endothelial growth factor expression by extracellular and intracellular pH. *Proceedings of the 89th Annual Meeting of American Association for Cancer Research* **39**: 378.

Young SD, Marshall RS and Hill RP (1988) Hypoxia induces DNA overreplication and enhances metastatic potential of murine tumor cells. *Proc Natl Acad Sci USA* **85**: 9533–9537

Zinn K, DiMaio D and Maniatis T (1983) Identification of two distinct regulatory regions adjacent to the human $\beta$-interferon gene. *Cell* **34**: 865–879

 *© 1999 Cancer Research Campaign*
